# P2y_12_ inhibitor monotherapy after 1–3 months dual antiplatelet therapy in patients with coronary artery disease and chronic kidney disease undergoing percutaneous coronary intervention: a meta-analysis of randomized controlled trials

**DOI:** 10.3389/fcvm.2023.1197161

**Published:** 2023-07-06

**Authors:** Yanqiao Yu, Deng Pan, Ruina Bai, Jinwen Luo, Yu Tan, Wenhui Duan, Dazhuo Shi

**Affiliations:** ^1^ Department of Graduate School, Beijing University of Chinese Medicine, Beijing, China; ^2^ Xiyuan Hospital, China Academy of Chinese Medical Sciences, Beijing, China; ^3^ National Clinical Research Center for Chinese Medicine Cardiology, Xiyuan Hospital, China Academy of Chinese Medical Sciences, Beijing, China

**Keywords:** P2Y_12_ inhibitor monotherapy, dual antiplatelet therapy, chronic kidney disease, coronary artery disease, meta-analysis

## Abstract

**Introduction:**

In patients with coronary artery disease (CAD) and chronic kidney disease (CKD) undergoing percutaneous coronary intervention (PCI), whether short-term dual antiplatelet therapy (DAPT) followed by P2Y_12_ inhibitors confers benefits compared with standard DAPT remains unclear. This study aimed to assess the efficacy and safety of 1–3 months of DAPT followed by P2Y_12_ monotherapy in patients with CAD and CKD undergoing PCI.

**Methods:**

PubMed, Embase, and the Cochrane Library were searched to identify randomized controlled trials (RCTs) comparing the P2Y_12_ inhibitor monotherapy after a 1–3 months DAPT vs. DAPT in patients with CAD and CKD after PCI. The primary outcome was the incidence of major adverse cardiovascular events (MACEs), defined as a composite of all-cause mortality, myocardial infarction, stent thrombosis, target-vessel revascularization, and stroke. The safety outcome was the major bleeding events, defined as a composite of TIMI major bleeding or Bleeding Academic Research and Consortium (BARC) type 2, 3, or 5 bleeding. The pooled risk ratios (RRs) with 95% confidence intervals (CIs) were calculated with a fixed- or random-effects model depending on the heterogeneity among studies.

**Results:**

Four RCTs including 20,468 patients (2,833 patients with CKD and 17,635 without CKD) comparing P2Y_12_ inhibitor monotherapy with DAPT were included in our meta-analysis. Patients with CAD and CKD had higher risk of ischemic and bleeding events. P2Y_12_ inhibitor monotherapy after 1–3 months of DAPT significantly reduced the risk of major bleeding compared to DAPT in CKD patients (RR: 0.69, 95% CI: 0.51–0.95, *P *= 0.02) and non-CKD patients (RR: 0.66, 95% CI: 0.49–0.89, *P *= 0.01). No significant difference regarding MACEs between P2Y_12_ inhibitor monotherapy and DAPT was found in CKD patients (RR: 0.88, 95% CI: 0.59–1.31, *P *= 0.53) and non-CKD (RR: 0.91, 95% CI: 0.79–1.04, *P *= 0.17).

**Conclusion:**

P2Y_12_ inhibitor monotherapy after 1–3 months of DAPT was an effective strategy for lowering major bleeding complications without increasing the risk of cardiovascular events in patients with CAD and CKD undergoing PCI as compared with DAPT

**Systematic review registration:**

https://www.crd.york.ac.uk/PROSPERO/, CRD42022355228.

## 1. Introduction

Dual antiplatelet therapy (DAPT) is the primary treatment for coronary artery disease (CAD) patients following percutaneous coronary intervention (PCI). DAPT exerts its effects by suppressing platelet activation and aggregation, thereby reducing ischemic cardiovascular events. However, a previous meta-analysis that investigated the effects of varying durations of DAPT after PCI found that standard DAPT, as compared to short-term DAPT, was generally associated with increased bleeding risk (1). Recent studies have also shown that short-duration P2Y_12_ inhibitor monotherapy, administered after 1–3 month of DAPT, resulted in lower bleeding rates and similar ischemic events when compared to 12 months or longer DAPT (2–4). Given the trade-off between ischemic and bleeding risks, de-escalation of DAPT duration has emerged as an alternative therapy (5), which is now recommended as an option in the current guidelines (6–8).

Patients with CAD and chronic kidney disease (CKD) face a higher risk of bleeding and ischemic events, which significantly impacts clinical prognosis (9). Thus, antiplatelet treatment for these patients requires extra caution to balance the risks of bleeding and ischemia. Despite the high prevalence of CAD and CKD coexistence, previous clinical trials rarely included these patients (10). Additionally, CKD patients experience bleeding events more frequently, especially severe events like intracranial hemorrhage, compared to those without CKD. Nonetheless, the optimal duration of DAPT for this high-risk subgroup remains unclear. Therefore, we conducted a meta-analysis to assess the safety and effectiveness of P2Y_12_ inhibitor monotherapy after 1–3 months of DAPT in patients with CAD and CKD.

## 2. Methods

This study strictly adheres to the guideline of Preferred Reporting Items for Systematic Reviews and Meta-Analyses (PRISMA) (11), and has already registered in the PROSPERO (Number: CRD42022355228).

### 2.1. Study selection

Two independent researchers (YQY and JWL) conducted PubMed, Embase, and the Cochrane Library searches to identify eligible studies from the inception of each database to September 1, 2022. The search items included “percutaneous coronary intervention”, “P2Y_12_ inhibitor monotherapy”, “dual antiplatelet”, “drug-eluting stent”, “randomized controlled trial” and “chronic kidney disease” in different combinations. The inclusion criteria of the studies are as follows (1): randomized controlled comparison between P2Y_12_ inhibitor monotherapy after a maximum of 3 months of DAPT vs. DAPT for at least 12 months (2); population includes CKD patients undergoing PCI with drug-eluting stents for stable CAD or ACS. Only publications in English were included. CKD was defined as an estimated glomerular filtration rate of less than 60 ml/min per 1.73 m^2^ of body-surface area. Studies for which the full text was unavailable or without sufficient valid data were excluded. Furthermore, we also reviewed the references of the included articles and the relevant review articles. The search details were provided in Supplementary Table S1.

### 2.2. Outcomes

The primary outcome was the incidence of major adverse cardiovascular events (MACEs), defined as a composite of all-cause mortality, myocardial infarction, stent thrombosis, target-vessel revascularization, or stroke at the individual trial protocol–defined follow-up. The safety outcome was the major bleeding events, defined as a composite of Bleeding Academic Research and Consortium (BARC) type 2, 3, or 5 bleeding, or thrombolysis in myocardial infarction (TIMI) major bleeding.

### 2.3. Data extraction and quality assessment

All analyses were performed independently on the data set reported in the subgroup analysis of each trial. Data concerning the publication year, the study type, the time point of randomization, the intervention strategy as well as the baseline characteristics of the patients were also extracted.

The methodological quality assessment of the included studies was determined using the Cochrane Collaboration risk-of-bias tool 2 (RoB 2) independently by two researchers (12). The third researcher would sort and make a final decision if there were any disagreements.

### 2.4. Statistical analysis

The results of treatment effects were combined with a Mantel–Haenzel fixed-effect model or DerSimonian–Laird random-effects model depending on the heterogeneity among studies and presented as risk ratios (RRs) with 95% confidence intervals (CIs) (13). A random-effect model was prespecified for *I*^2^ statistic ≥50%, and a fixed-effect model would be used when *I*^2^ < 50%. *I*^2^ is calculated quantitatively in case of significant heterogeneity, and *I*^2^ > 50% indicates a notable heterogeneity. The common heterogeneity between the trials was assessed qualitatively using the Cochran's *Q* statistic, with *P*_Heterogeneity_ <0.05 indicating significant heterogeneity. A two-sided *P*-value of <0.05 was considered statistically significant. Sensitivity analysis was performed by removing one study at a time to confirm that any individual study did not drive our findings. All statistical analyses were conducted using R software (Version 4.0.5) with the “meta” package.

## 3. Results

**Figure 1:**
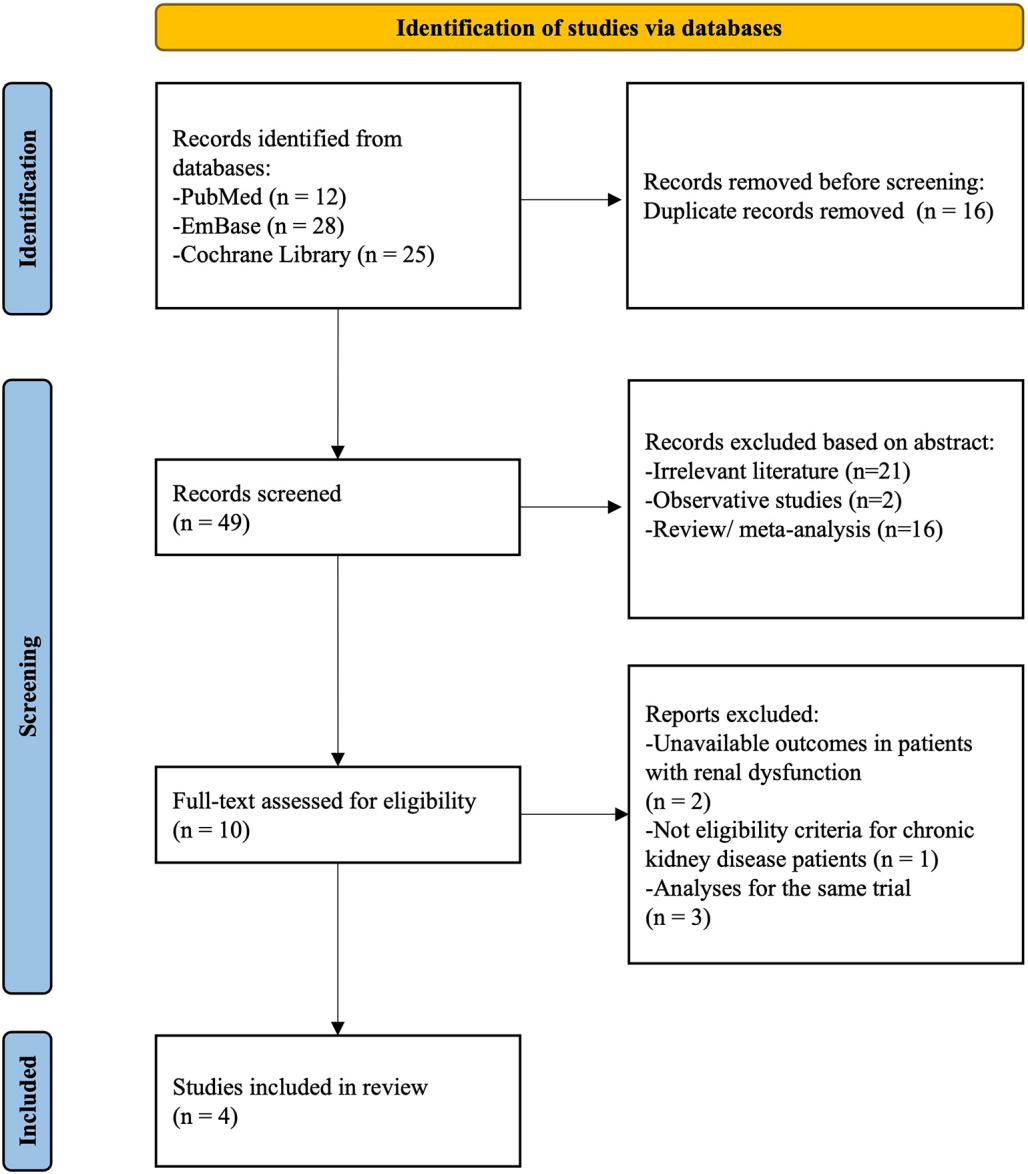
Flowchart of the study selection progress.

The initial literature research screened 65 articles from the PubMed, Embase, and Cochrane library databases. Out of these, 49 articles were screened, and ten full-text articles were retrieved and assessed for eligibility. Patients from STOP-DAPT2 trial (2) were excluded due to the unavailability of component outcomes such as major adverse cardiovascular events (MACE) and bleeding events, which are necessary to calculate study power. Finally, four studies fulfilled the inclusion criteria and were included in the meta-analysis (14–17) (Figure 1). We used the outcome data extracted from the subgroup analysis or substudy of the major trials. A total of 20,468 patients (2,833 CKD and 17,635 non-CKD) were primarily analyzed. Of the CKD patients, 1,400 patients (49.4%) were treated with P2Y_12_ inhibitor monotherapy and 1,433 patients (50.6%) with DAPT. Among the four studies, the TICO trial (15) only enrolled ACS patients, and the other three enrolled ACS and stable CAD patients. The SMART-CHOICE trial (14) used aspirin and one P2Y_12_ inhibitor (clopidogrel, ticagrelor, or prasugrel) for DAPT, while the other trials used aspirin and ticagrelor. The baseline characteristics and the definition of the MACEs and major bleeding for the individual included studies are shown in Table 1.

**Table 1:** Baseline characteristics of coronary kidney disease patients in the included studies.

Study	SMART-CHOICE (14)		TICO (15)		TWILIGHT (16)		GLASSY (17)	
Population	ACS or stable CAD undergoing PCI		ACS undergoing PCI		Post PCI with a high risk of ischemic or bleeding events		ACS or stable CAD undergoing PCI	
Randomization	At index PCI		At index PCI		3 months after PCI		At index PCI	
Follow-up	12 months		12 months		15 months (12 months after randomization)		24 months	
Study design	RCT		RCT		RCT		RCT	
Arm	P2Y_12_ inhibitor	DAPT	P2Y_12_ inhibitor	DAPT	P2Y_12_ inhibitor	DAPT	P2Y_12_ inhibitor	DAPT
Age (mean)	64.6	64.4	61	61	65.2 ± 10.3	65.1 ± 10.4	64.9 ± 10.3	64.8 ± 10.3
Intervention	Aspirin + P2Y_12_ inhibitor for 3 months, followed by P2Y_12_ inhibitor monotherapy for 9 months	Aspirin + P2Y_12_ inhibitor for 12 months	DAPT for 3 months, followed by ticagrelor monotherapy for 9 months	Aspirin + ticagrelor for 12 months	Ticagrelor + aspirin for 3 months, followed by ticagrelor monotherapy for 12 months	Ticagrelor + aspirin for 15 months	Aspirin + ticagrelor for 1 month, followed by ticagrelor monotherapy for 23 months	Aspirin + ticagrelor for 12 months, followed by aspirin for 12 months
CKD, *n* (%)	97 (3.2)		620 (20.3)		1,111 (16.3)		1,005 (13.2)	
Efficacy endpoint	Death, MI, or stroke		Death, MI, stent thrombosis, stroke, and target-vessel revascularization		Death, MI, and stroke		Death, MI, stroke, and target-vessel revascularization	
Safety endpoint	BARC 2–5 type bleeding		TIMI major bleeding		BARC 2, 3 or 5 type bleeding		BARC 3 or 5 type bleeding	

ACS, acute coronary syndrome; BARC, Bleeding Academy Research Consortium; CAD, coronary artery disease; CKD, chronic kidney disease; DAPT, dual antiplatelet therapy; MI, myocardial infarction; PCI, percutaneous coronary intervention; RCT, randomized controlled trial; TIMI, thrombolysis in myocardial infarction.

The results of the risk of bias assessment with the RoB 2 tool are summarized in Supplementary Figure S1. Three studies were considered at high risk for overall risk of bias, and the TWILIGHT trial (16) presented only unclear risk for overall risk of bias. All included trials were open-label RCTs except for the TWILIGHT trial (16), which was double-blinded.

### 3.1. Primary outcome

**Figure 2:**
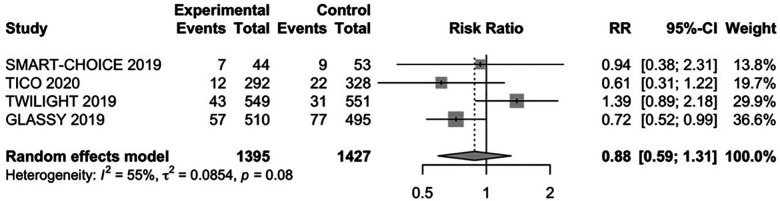
The RR of primary outcome for patients with CAD and CKD treated with P2Y_12_ inhibitor monotherapy after 1–3 months DAPT vs DAPT. CI, confidence interval; RR, risk ratio.

Among all enrolled patients, the primary outcome occurred higher in CKD patients after PCI than those without CKD (8.53% vs. 4.11%). The primary outcome of patients with CKD and without CKD are shown in Figure 2 and Supplementary Figure S2. In patients with CAD and CKD, the primary outcome of MACEs occurred in 119 (8.47%) patients with P2Y_12_ inhibitor monotherapy and 139 (9.65%) patients with DAPT. There were no significant differences for MACEs (RR: 0.88, 95% CI: 0.59–1.31, *P *= 0.53, *I*^2 ^= 55%, *P*_Heterogeneity _= 0.08) between the P2Y_12_ inhibitor monotherapy and DAPT strategy. In non-CKD patients, the primary outcome of MACEs occurred in 362 (4.11%) patients with P2Y_12_ inhibitor monotherapy and 398 (4.54%) patients with DAPT. P2Y12 inhibitor monotherapy had a similar risk of MACEs compared to DAPT (RR: 0.91, 95% CI: 0.79–1.04, *P *= 0.17, *I*^2 ^= 0, *P*_Heterogeneity _= 0.50). The risk of MACEs in non-CKD patients receiving P2Y_12_ inhibitor monotherapy was numerically but not significantly lower compared with DAPT.

### 3.2. Safety outcome

**Figure 3:**
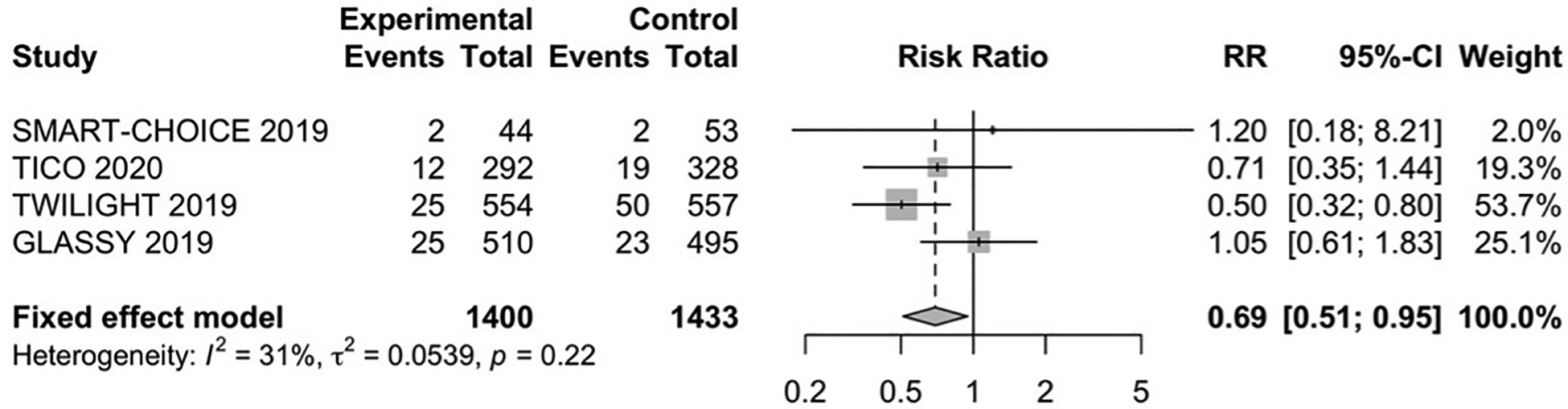
The RR of safety outcome for patients with CAD and CKD treated with P2Y_12_ monotherapy after 1–3 months DAPT vs DAPT. CI, confidence interval; RR, risk ratio.

The major bleeding complications were defined according to the TIMI hemorrhage classification in study TICO (15) and the BARC definition for the other studies. The primary outcome occurred higher in CKD patients after PCI than those without CKD (4.57% vs. 2.50%). The endpoint of major bleeding occurred in 64 (4.56%) and 221 (2.50%) patients with P2Y_12_ inhibitor monotherapy, and 94 (6.54%) and 332 (3.77%) patients with DAPT in CKD and non-CKD patients, respectively. We found that 1–3 months of DAPT followed by P2Y_12_ inhibitor monotherapy had a lower risk of major bleeding compared with those applying DAPT in patients with CAD and CKD (RR: 0.69, 95% CI: 0.51–0.95, *P *= 0.02, *I*^2 ^= 31%, *P*_Heterogeneity _= 0.22) and non-CKD (RR: 0.66, 95% CI: 0.49–0.89, *P *= 0.01, *P*_Heterogeneity _= 0.06) with low evidence of heterogeneity among studies (Figure 3; Supplementary Figure S3).

### 3.3. Publication bias and sensitivity analysis

We did not conduct funnel plots to assess for selection bias owing to the relatively small number of included studies, thereby limiting the test power of our meta-analysis. The individual trial influences on the primary and safety outcomes did not reveal inconsistency (Supplementary Figure S4).

## 4. Discussion

Our meta-analysis showed that CKD patients undergoing PCI indeed had a higher risk of ischemic and bleeding events than those without CKD. P2Y_12_ inhibitor monotherapy after 1–3 months of DAPT was associated with a significantly lower risk of major bleeding than DAPT, and the magnitude of this effect was consistent among patients with and without CKD. Nevertheless, we did not detect evidence of a benefit from 1 to 3 months of DAPT followed by P2Y_12_ inhibitor monotherapy with respect to MACEs compared with DAPT among these patients, irrespective of the presentation of renal dysfunction. To the best of our knowledge, this is the first meta-analysis that compared the pooled efficacy and safety of P2Y_12_ receptor inhibitor monotherapy with DAPT among individuals with CAD and CKD undergoing PCI.

The present meta-analysis included large-scale randomized controlled trials (RCTs) investigating the efficacy and safety of short-term DAPT in reducing bleeding events without increasing MACEs in patients with CAD undergoing PCI (3, 14–16). This is consistent with several previous meta-analyses, which concluded that short-term DAPT may have superiority with respect to the safety and similar efficacy compared to standard 12-month DAPT regimen, regardless of comorbidity in the general population with CAD (18, 19). Similarly, previous trials exclusively enrolling high bleeding risk patients also reported the benefits of short-term DAPT (20). Our study found that P2Y_12_ inhibitor monotherapy after 1–3 months of DAPT after PCI was not associated with potential harm. When compared to DAPT, P2Y_12_ inhibitor monotherapy significantly reduced the risk of major bleeding and demonstrated comparable rates of MACEs. The magnitude of this effect was consistent among both CKD and non-CKD patients undergoing PCI.

CKD patients are associated with poor prognoses on cardiovascular outcomes in line with their more complex coronary artery lesion characteristics and hypercoagulable state (21, 22). Concerns over a shortened DAPT regimen in these patients are based on the heterogeneity of the clinical, anatomic, and biochemical factors as compared to general CAD patients. It is also worth mentioning that CKD patients exhibit an increased risk of high on-treatment platelet reactivity than non-CKD patients (23, 24), which increases the risk of ischemic events after PCI. Notably, the risk of ischemia is positively correlated with the severity of renal dysfunction, as shown in a *post hoc* analysis of the EPICOR trial (25). On the other hand, CKD patients reveal platelet activation, aggregation, and adhesion dysfunctions, which contribute to hemorrhage (26). Individuals with CKD have a higher risk of bleeding, and the bleeding prevalence increases with worsening renal function (27). Implementing an antiplatelet regimen that maximizes efficacy and safety in CAD patients with CKD remains crucial in this situation.

Aspirin in combination with a P2Y_12_ inhibitor represents the cornerstone therapy after stent implantation and has been well-established. However, there is a strong trend toward worse outcomes in patients with CAD and CKD. In a previous pooled analysis of five RCTs involving 1,273 CKD patients, the incidence of ischemic events was significantly higher among those who received aspirin than those who received DAPT, regardless of the DAPT duration (28). The reasons for this may be multifactorial, potentially including the influence of deteriorating renal function, altered pharmacokinetics, and high on-treatment platelet reactivity in CKD patients when treated with aspirin (29–31). Unfortunately, there is a scarcity of studies investigating the optimal antiplatelet regimen and duration for CKD patients after PCI.

The choice of post-DAPT monotherapies has been explored before, with recent evidence highlighting the potential benefits of P2Y_12_ inhibitor monotherapy. The HOST-EXAM (Harmonizing Optimal Strategy for Treatment of Coronary Artery Stenosis–Extended Antiplatelet Monotherapy) trial (32) showed that clopidogrel monotherapy significantly reduced both thrombotic (HR: 0.68, 95% CI: 0.52–0.87) and bleeding events (HR: 0.70; 95% CI: 0.51–0.98) at 12 months compared with the aspirin monotherapy. And its extended study, the HOST-EXAM Extended study (33), further supported these findings, demonstrating a similar reduction with no significant difference in all-cause mortality over a median follow-up of 5.8 years.

Regardless of the concerns on the increased risk of gastrointestinal bleeding associated with aspirin, the antithrombotic properties of novel potent P2Y12 inhibitors are superior to aspirin, as demonstrated by a meta-analysis of 42,108 atherosclerotic patients, which yielded a lower risk of MI when comparing the antithrombotic effects between aspirin and P2Y_12_ inhibitor monotherapy (OR: 0.81; 95% CI: 0.66–0.99) (34). Furthermore, P2Y_12_ inhibitors have been shown to reduce the bleeding rate compared to DAPT (35). In the present study, 1–3 months of DAPT followed by P2Y_12_ inhibitor monotherapy was superior to DAPT in preventing bleeding complications. A pooled analysis of the SMART-DATE and SMART-CHOICE trials suggested a trend toward a lower risk of major bleeding events with P2Y12 inhibitor monotherapy after a short duration of DAPT compared to standard DAPT and aspirin monotherapy after short-DAPT (36). A similar result for major bleeding with short-term DAPT followed by aspirin monotherapy compared with standard DAPT in CKD patients was previously suggested by a meta-analysis (RR: 0.69; 95% CI: 0.30–1.60, *P *= 0.39) (37). Our result is in line with the previous evidence but suggests a clear benefit of decreased major bleeding events. The withdrawal of aspirin and less injury to gastric mucosal by P2Y_12_ inhibitor monotherapy might explain our result (38).

Several lines of evidence have already suggested that P2Y_12_ inhibitor monotherapy might provide similar ischemic benefits to standard DAPT (35, 39). The additional use of aspirin does not further contribute to excess anti-platelet capability than that of P2Y_12_ antagonists alone (40). The MATCH trial (Molecular Analysis for Therapy Choice) (41) indicated that in high-risk patients with recent ischemic stroke, the DAPT regimen with clopidogrel and aspirin did not lead to a significant decrease in ischemic events compared to clopidogrel monotherapy.

Considering the clinical situations where a considerable proportion of patients undergoing PCI need to discontinue DAPT due to bleeding complications, especially in the context of an aging population with comorbidities, there is a growing interest in conducting clinical trials to evaluate the effectiveness of a shortened duration of DAPT in high bleeding risk (HBR) patients. Recent trials have focused on HBR patients receiving DAPT after PCI and investigated whether a shorter duration of DAPT, compared to the standard duration, could lead to improved outcomes. These trials consistently demonstrated favorable results, indicating that a shorter duration of DAPT was associated with a reduced risk of bleeding events while maintaining similar rates of ischemic events (42, 43). The MASTER DAPT (Management of High Bleeding Risk Patients Post Bioresorbable Polymer Coated Stent Implantation with an Abbreviated vs. Standard DAPT Regimen) trial (44) which involved 4,434 CAD patients at HBR indicated that short duration of DAPT was non-noninferiority to standard DAPT with regard to net clinical events and major adverse cardiac or cerebral events, while short duration of DAPT resulted in a lower incidence of bleeding events.

When it comes to CKD patients, whether the risk factor of renal dysfunction attenuates the clinical efficacy of P2Y_12_ inhibitor remains to be investigated. Our study suggests a comparable risk in MACE in individuals with CAD and CKD when comparing P2Y_12_ inhibitor monotherapy with standard DAPT. The pharmacokinetic and pharmacodynamic properties of the active metabolite of P2Y_12_ inhibitors are similar in CKD and non-CKD patients, with a marginal difference in the anti-platelet effect (45). Therefore, 1–3 months of DAPT followed by a P2Y_12_ inhibitor may be a reasonable strategy.

## 5. Limitation

Our study has several limitations. Firstly, we included a relatively small number of studies, thereby preventing us from conducting subgroup analysis by the concrete P2Y_12_ inhibitor type. In the PLATO (Platelet Inhibition and Patient Outcomes) trial, ticagrelor was associated with a 16% lower rate of MACE in high-risk ACS patients when compared to clopidogrel (46). The subgroup analysis found that, in CKD patients undergoing PCI for ACS, ticagrelor was associated with a larger benefit on anti-ischemic without significantly increasing the bleeding risks on top of clopidogrel (47). In our study, the sensitivity analysis showed that removing SMART-CHOICE trial (14) was consistent with the initial analysis for all outcomes (Supplementary Figure S4), implying that the type of P2Y_12_ inhibitors may not affect the incidence of ischemic and bleeding events in patients with CAD and CKD. Future studies are still warranted to analyze the effects of different P2Y_12_ inhibitors. Secondly, the definitions of the bleeding and ischemic endpoint were slightly different among the included studies, which may dilute the reliability of our result. Thirdly, the inherent constraints of the included studies place restrictions on our meta-analysis. The outcome data regarding the comparisons of P2Y_12_ inhibitors and DAPT in patients with CAD and CKD undergoing PCI are only available in the subgroup analysis of the involving RCTs. Hence our meta-analysis was conducted on a trial level, and we failed to consider the risks of a patient level. For this reason, we failed to evaluate the impact of CKD severity and did not stratify patients based on their baseline clinical presentation such as ACS vs. chronic coronary disease and coexisting comorbidities. Despite the possibility that the absence of the CKD stage affected the treatment effect, the small size of each subgroup made it difficult for individual components to identify heterogeneity. Due to the limited availability of data, we were unable to address this specific analysis. Further research exploring this distinction is warranted.

## 6. Conclusion

Our data provide the best estimates to date of the risks and benefits of P2Y_12_ monotherapy after 1–3 months of DAPT in the setting of patients with CAD and CKD. On the basis of our analysis, 1–3 months of DAPT followed by P2Y_12_ inhibitor monotherapy might be a promising strategy in these patients with significantly lower bleeding complications and a reduction trend in ischemic events compared with standard DAPT. Large-scale studies with high quality and adequate power to estimate its efficacy and safety are warranted.

## Data Availability

The original contributions presented in the study are included in the article/**Supplementary Material**, further inquiries can be directed to the corresponding authors.
