# Supplementary material for: P2y12 inhibitor monotherapy after 1–3 months dual antiplatelet therapy in patients with coronary artery disease and chronic kidney disease undergoing percutaneous coronary intervention: a meta-analysis of randomized controlled trials
Source: Front Cardiovasc Med. 2023 Jul 6;10:1197161. doi: 10.3389/fcvm.2023.1197161 (PMC10357506; doi:10.3389/fcvm.2023.1197161)
Supplement: Supplementary file 1 [file Datasheet1.docx]

Supplementary Material

P2Y_12_ inhibitor monotherapy after 1-3 months dual antiplatelet therapy in patients with coronary artery disease and chronic kidney disease undergoing percutaneous coronary intervention: A meta-analysis of randomized controlled trials

Yanqiao Yu ^1,2^, Deng Pan ^1,2^, Ruina Bai ^3^, Jinwen Luo ^2^, Yu Tan ^2^, Wenhui Duan^3*^, Dazhuo Shi^3*^

*** Correspondence:** Corresponding Author: Wenhui Duan (duanwh168@126.com) and Dazhuo Shi (shidazhuo@126.com).

# Supplementary Tables

**Supplementary Table 1. Search strategy.**

| **PubMed** | | |
| --- | --- | --- |
| **Search number** | **Query** | **Results** |
| **#4** | #1 AND #2 AND #3 | 938 |
| **#3** | (randomized controlled trial[Publication Type]) OR (controlled clinical study[Publication Type]) | 696,496 |
| **#2** | (percutaneous coronary intervention[Title/Abstract]) OR (drug-eluting stent[Title/Abstract]) | 43,619 |
| **#1** | (dual antiplatelet therapy[Title/Abstract]) OR (P2Y12 inhibitor[Title/Abstract]) OR (clopidogrel[Title/Abstract]) OR (ticagrelor[Title/Abstract]) OR (prasugrel[Title/Abstract]) | 19,268 |
| **Embase** | | |
| **Search number** | **Query** | **Results** |
| **#12** | #7 AND #10 AND #11 | 1543 |
| **#11** | #3 OR #4 | 41147 |
| **#10** | #8 OR #9 | 834123 |
| **#9** | 'randomized controlled trial'/exp | 726511 |
| **#8** | 'randomized controlled trial':ab,ti OR 'clinical study':ab,ti OR 'controlled clinical trial':ab,ti | 229549 |
| **#7** | #5 OR #6 | 144504 |
| **#6** | 'percutaneous coronary intervention'/exp OR 'drug eluting stent'/exp | 138632 |
| **#5** | 'percutaneous coronary intervention':ti,ab,kw OR 'drug eluting stent':ti,ab,kw | 75192 |
| **#4** | ticagrelor:ti,ab,kw OR clopidogrel:ti,ab,kw OR 'purinergic p2y12 receptor':ti,ab,kw | 29169 |
| **#3** | #1 OR #2 | 17318 |
| **#2** | 'dual antiplatelet therapy'/exp | 11576 |
| **#1** | 'dual antiplatelet therapy':ti,ab,kw | 10582 |
| **Cochrane Library** | | |
| **Search number** | **Search** | **Results** |
| **#4** | #1 AND #2 AND #3 | 1330 |
| **#3** | ("percutaneous coronary intervention"):ti,ab,kw OR ("drug eluting stent"):ti,ab,kw (Word variations have been searched) | 13259 |
| **#2** | ("randomized controlled trial"):pt OR ("randomized clinical trial"):pt (Word variations have been searched) | 556044 |
| **#1** | (dual antiplatelet therapy):ti,ab,kw OR (prasugrel):ti,ab,kw OR (ticagrelor):ti,ab,kw OR ("clopidogrel"):ti,ab,kw OR (P2Y12 inhibitor):ti,ab,kw (Word variations have been searched) | 7934 |

## Supplementary Figures


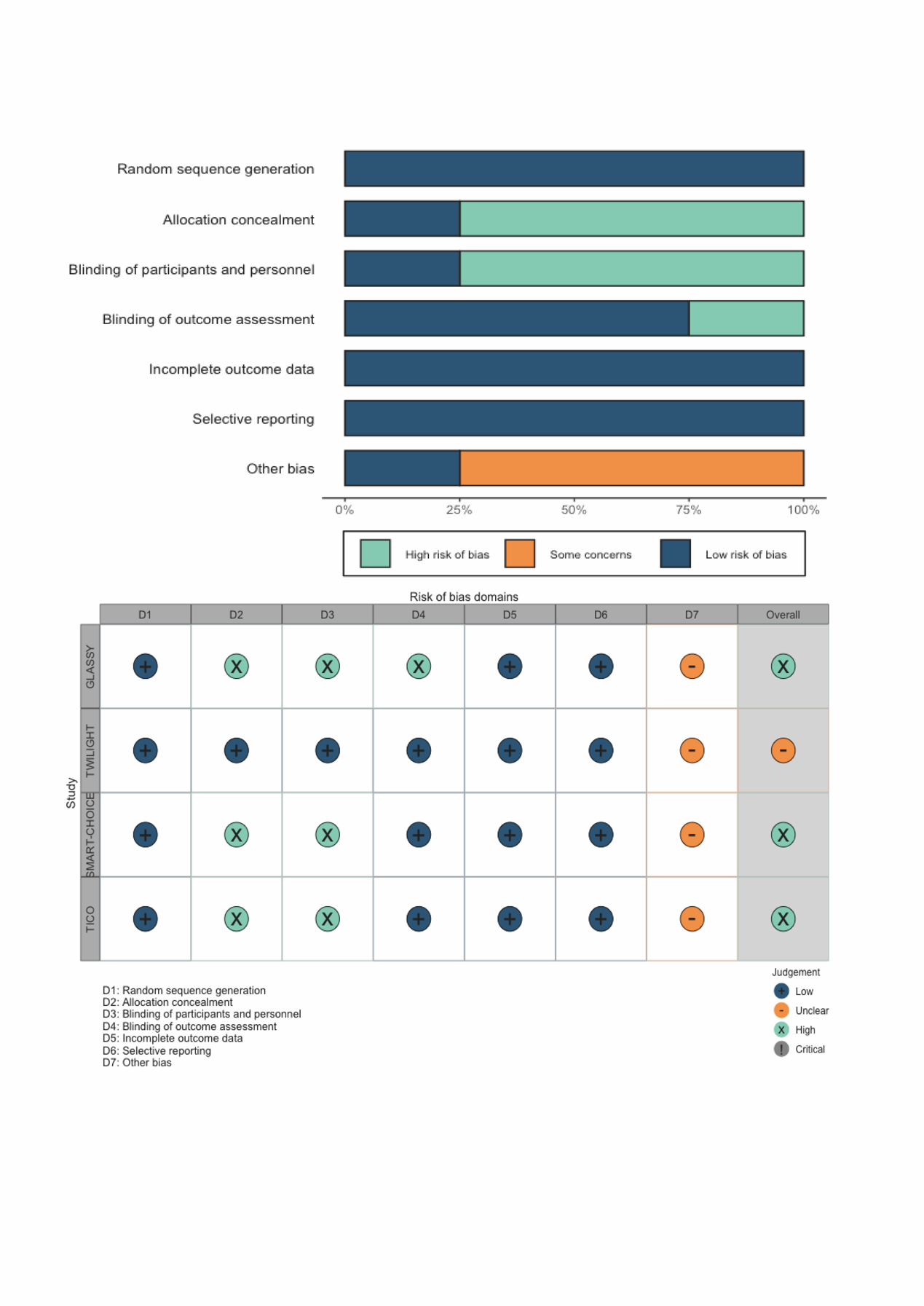


**Supplementary Figure 1. Qualitative assessment of potential sources of bias across the included trials.**

**
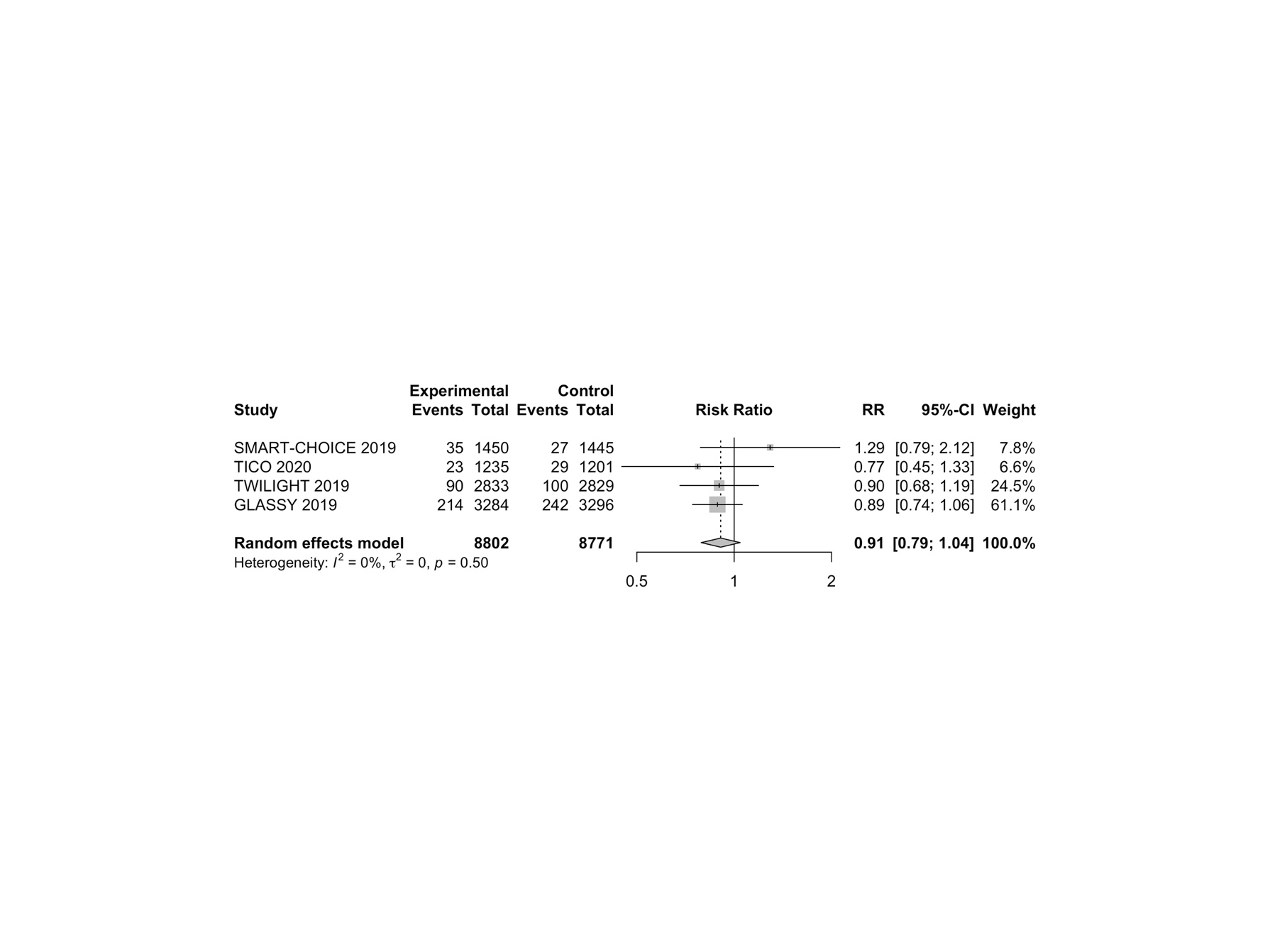
**

**Supplementary Figure 2.** **The RR of primary outcome for** **patients without CKD treated with P2Y_12_ monotherapy after 1-3 months DAPT versus DAPT.** CI: confidence interval; RR, risk ratio.


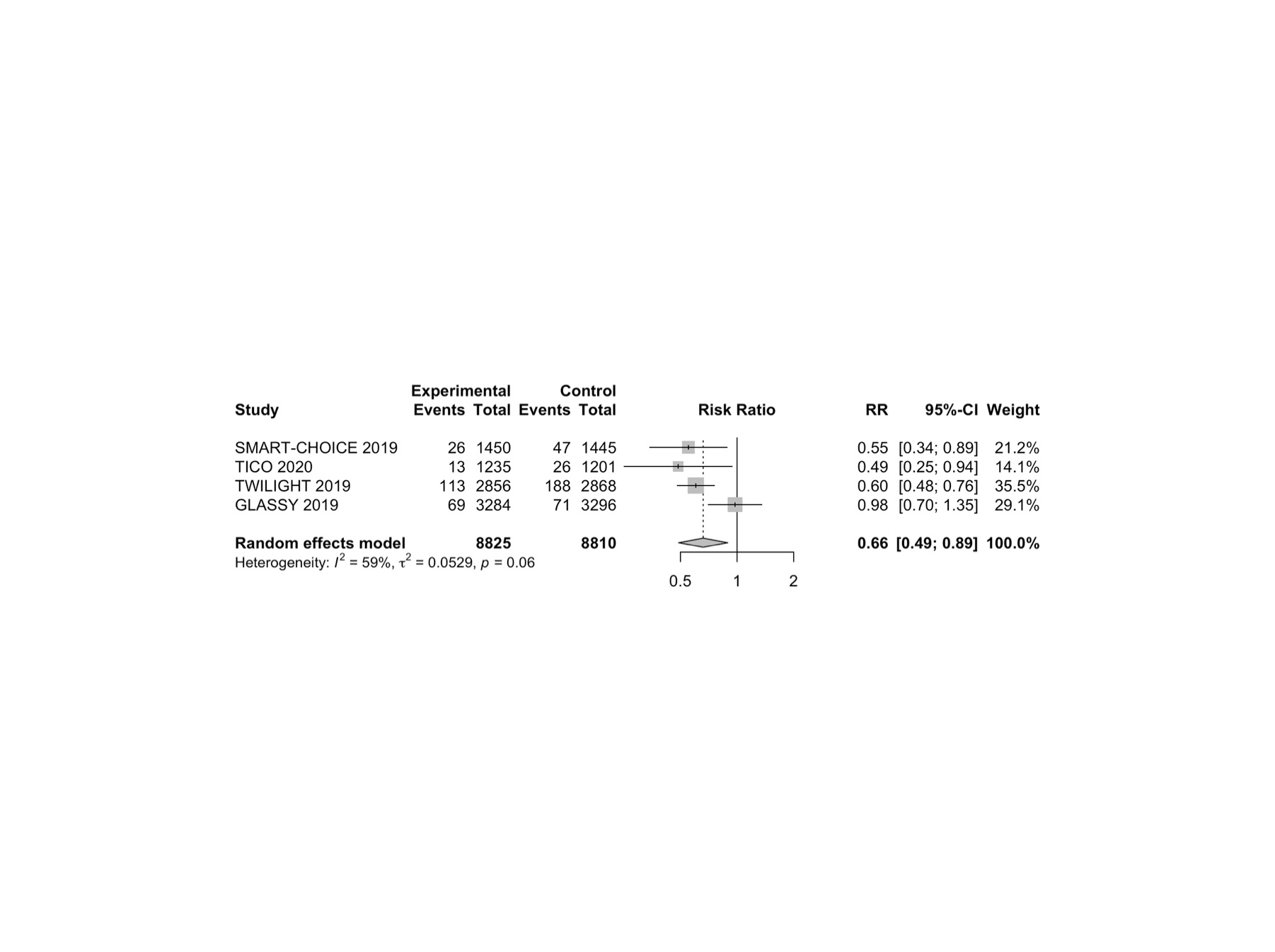


**Supplementary Figure 3. The RR of safety outcome for patients without CKD treated with P2Y_12_ monotherapy after 1-3 months DAPT versus DAPT.** CI: confidence interval; RR, risk ratio.

**

**

**Supplementary Figure 4. The sensitivity analysis of the primary outcome and the safety outcome. (A) primary outcome; (B) safety outcome.**
